# Application of nisin as a potential drug candidate for electrochemotherapy

**DOI:** 10.3389/fonc.2025.1689261

**Published:** 2025-11-28

**Authors:** Olga Michel, Barbora Lekešytė, Veronika Malyško-Ptašinskė, Arnoldas Morozas, Paulina Malakauskaitė, Eglė Mickevičiūtė-Zinkuvienė, Augustinas Želvys, Justinas Ivaška, Julita Kulbacka, Vitalij Novickij

**Affiliations:** 1 Department of Molecular and Cellular Biology, Faculty of Pharmacy, Wroclaw Medical University, Wrocław, Poland; 2 Department of Immunology and Bioelectrochemistry, State Research Institute Centre of Innovative Medicine, Vilnius, Lithuania; 3 Faculty of Electronics, Vilnius Gediminas Technical University, Vilnius, Lithuania; 4 Faculty of Medicine, Vilnius University, Vilnius, Lithuania

**Keywords:** nisin, nsPEF, µsPEF, electroporation, breast cancer, ECT

## Abstract

**Introduction:**

Electroporation (EP) is a technique that transiently increases the permeability of the cell membrane through the application of high-voltage electric pulses, facilitating the intracellular delivery of therapeutic agents or the selective ablation of cells. Combination of EP with cytotoxic drugs—most commonly bleomycin or cisplatin—is termed electrochemotherapy (ECT), which markedly enhances drug efficacy and permits targeted, locally controlled treatment with reduced systemic exposure. Currently, in addition to microsecond (µs) pulses, nanosecond (ns) pulses are being proposed for clinical use to mitigate certain ECT-associated side effects. However, achieving robust permeabilization with nsPEF typically requires higher electric fields. Nisin is a polycyclic antibacterial peptide with anticancer potential that can be leveraged in this context.

**Methods:**

To date, the permeabilizing properties of nisin have been employed alongside an external electric field exclusively in bacterial systems and artificial membranes. In this study, we investigated the impact of nisin on membrane permeabilization, resealing, and viability of 4T1 breast cancer cells exposed to microsecond and nanosecond electric pulses of varying field strengths and pulse frequencies.

**Results:**

Across all experimental conditions, nisin reduced the threshold voltage necessary for effective permeabilization and increased treatment-induced cell mortality.

**Discussion:**

Since nisin is non-toxic by itself, it represents a promising candidate for electrochemotherapy, potentially supporting its wider clinical application in the future.

## Highlights

- Nisin is a lantibiotic that interacts with membranes but is new to ECT use.
- PEF-induced permeabilization to YO-PRO-1 was greatly enhanced in nisin’s presence.
- Subtoxic nisin doses greatly reduced viability of electroporated cells.
- The effect was seen across all tested pulse lengths and frequencies.
- Nisin emerges as a novel candidate for either standalone or adjuvant ECT agent.

## 1 Introduction

**Figure 1:**
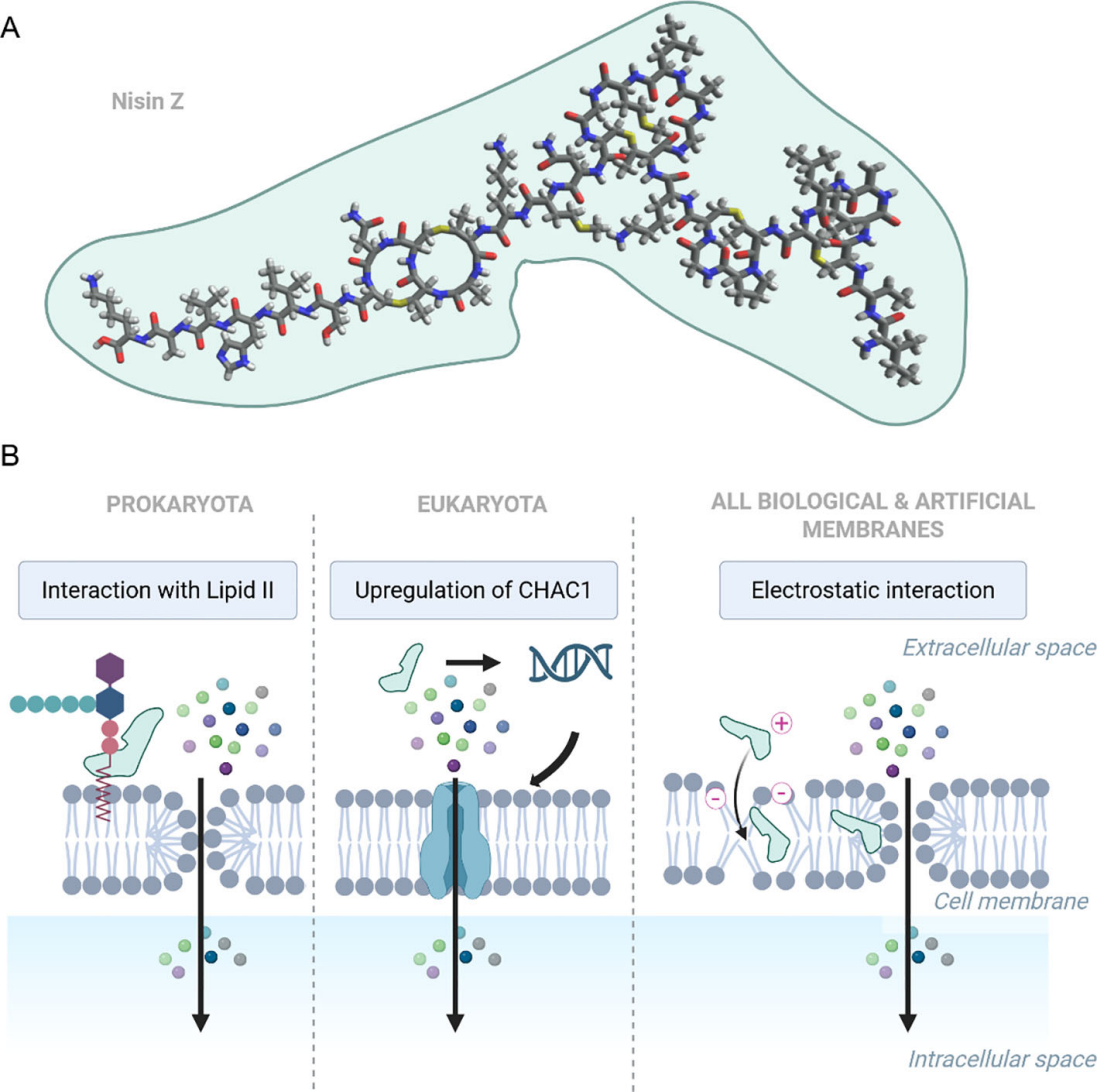
**(A)** Chemical structure of nisin Z (PubChem CID: 155489899), visualized using Avogadro software. **(B)** Representative mechanisms of nisin impact on membrane permeability found in various models: i) binding to Lipid II and assembling transmembrane pores (Prokaryota); ii) upregulation of CHAC1, disrupting redox and ion balance (Eukaryota), iii) electrostatic interactions destabilizing lipid bilayers, increasing permeability (found in biological and artificial membranes). Created in https://BioRender.com.

Nisin is a Type A lantibiotic produced by certain Gram-positive bacteria, first identified in 1928 by Rogers and Whittier in fermented milk cultures (1). Owing to its broad-spectrum antimicrobial efficacy and stability under various food processing conditions (2–4), nisin has been evaluated and approved for use as a food additive by major international health and regulatory agencies, including the World Health Organization (WHO) (5), European Food Safety Authority (EFSA) (6), and is recognized as Generally Recognized as Safe (GRAS) by the U.S. Food and Drug Administration (FDA) (7). Among all naturally occurring variants, nisin A and nisin Z are the most extensively studied (8, 9). Both variants exhibit very similar antimicrobial activity (10); however, the substitution of histidine with asparagine at position 27 in nisin Z enhances its diffusion capacity and solubility at neutral pH (11). The chemical structure of nisin Z is shown in **Figure 1A**.

Nisin has been demonstrated to exert various effects on both biological and artificial membranes (**Figure 1B**). Its most well-characterized mechanism involves targeting lipid II, a membrane-bound peptidoglycan precursor, which it utilizes as an anchor to inhibit cell wall biosynthesis and induce pores in the cytoplasmic membrane (12–15). A distinct mode of action has been detected in head and neck squamous cell carcinoma (HNSCC) cells, in which nisin highly upregulated the γ-glutamyl cyclotransferase (CHAC1), a known regulator of cation transport and apoptosis (16). Additionally, studies using artificial membranes have shown that nisin can exert permeabilizing effects independently of cellular machinery. In this context, the cationic peptide is electrostatically attracted to negatively charged phospholipids; upon insertion into the bilayer, it oligomerizes to form transient, nonspecific pores (17–20).

The concept of the nisin application in anticancer protocols was first explored by Maher and McClean (2006), who demonstrated its activity against two human colorectal adenocarcinoma cell lines. However, the effective concentrations required for anticancer activity were significantly higher than those needed for its antimicrobial effects (21). Ever since, the anticancer activity of nisin has been confirmed in several other cancer types (22) evoking apoptosis both *in vitro* (23–25) and *in vivo* (16, 26). Growing evidence suggests that nisin can inhibit tumor growth and selectively exert cytotoxic effects on cancer cells (9). Azmi et al. (2021) identified three key factors contributing to nisin’s selectivity: i) negative surface charge: cancer cell membranes are more negatively charged due to loss of phospholipid symmetry, attracting the positively charged nisin; ii) increased membrane fluidity: cancer cells have more fluid and unstable membranes, enhancing nisin’s ability to bind and disrupt them; iii) abundant microvilli: the high density of microvilli on cancer cells increases the surface area for nisin interaction (22). Altogether, it can be concluded that, unlike traditional chemotherapeutic agents, nisin exerts its effects through interactions with the cell surface rather than inducing DNA damage. Regardless of whether it acts through lipid II binding, gene upregulation, or direct electrostatic interactions, the mechanisms of nisin’s action on membranes ultimately converge on the disruption of ionic homeostasis, eventually leading to cell death.

The permeabilization effect of nisin resembles the phenomenon observed in cells exposed to pulsed electric fields (PEF). Applying an external voltage to cells positioned between electrodes results in pulsed electric field, which induces cell transmembrane potential (TMP) (27). If the TMP surpasses a critical threshold, hydrophilic pores form in the cell membrane - a process known as electroporation (EP) (28). Depending on the intensity of the applied electric field, this process can be either irreversible or reversible (29). The latter is used in a treatment modality called electrochemotherapy (ECT), in which PEFs are applied to enhance the transmembrane delivery of chemotherapeutic drugs (30, 31). Current applications of ECT in cancer treatment adhere to the ESOPE protocol, which utilizes microsecond pulsed electric field (µsPEF) to enhance the intracellular uptake of bleomycin, cisplatin or calcium (32). Despite its advantages, the application of ECT is still associated with several drawbacks including pain (33), muscle contractions (32), the need for repeated procedures (34), and profound tissue necrosis (35). A partial solution to these issues has been achieved by shortening the duration of the electric pulse to a few microseconds. For example, high-frequency microsecond pulses (H-FIRE) have been shown to reduce muscle contractions and pain compared with conventional IRE (36, 37). However, even with H-FIRE, some residual stimulation persists.

Further shortening the pulse duration to nanoseconds provides additional benefits. With ns pulsed electric fields (nsPEFs), the plasma membrane does not fully charge (a regime often termed supra-electroporation)-which means that higher field amplitudes are required to achieve permeabilization comparable to longer pulses. Nevertheless, skeletal and cardiac excitation is even lower than in H-FIRE, improving safety and tolerability (38, 39). At the same time, when ns pulses are delivered at high repetition frequencies, the plasma membrane cannot completely discharge between successive pulses. When the interpulse interval approaches the membrane charging constant, residual TMP accumulation occurs, leading to a gradual increase in the effective TMP (40–43). As a result, pulse trains delivered in the hundreds of kilohertz (kHz) to megahertz (MHz) range can enhance membrane permeabilization and lower the effective electroporation threshold (42, 44, 45). Early studies on high-frequency nsPEF have shown that pulse compression into the MHz range lowers excitation thresholds and facilitates electroporation (42). Such high-frequency bursts also help mitigate impedance effects, promoting a more uniform electric field distribution within the tumor due to the frequency dependence of bioimpedance (46). Importantly, nsPEF offer distinct biological mode of action by reaching intracellular targets - they can permeabilize internal membranes and trigger Ca^2+^ release, mitochondrial depolarization, reactive oxygen species (ROS) signaling, and regulated cell death - effects that are weak or absent with standard μsPEF application (47, 48). Expanding electroporation to include intracellular organelles and modulating cellular stress responses represent a rational approach to enlarge the effective treatment zone and enhance cytotoxicity when ablation is incomplete, as sometimes observed with H-FIRE (49, 50).

The main drawback with nsPEF application is that high voltages required to induce electroporation often exceed the operational limits of most nsPEF generators and pose significant high-voltage safety risks, thereby limiting the clinical applicability of submicrosecond pulses. In this study, we propose the use of nisin to lower the threshold electric field required for effective membrane permeabilization. Given that multiple studies report greater toxicity of nisin toward cancer cells than normal cells, combining nisin with pulsed electric fields provides an additional, localized selectivity mechanism that may reduce off-target cytotoxicity of ECT treatment. Moreover, transmembrane delivery via electroporation could further enhance nisin’s selectivity and anticancer activity. Finally, enhancing the controllability and efficacy of various PEF modalities could significantly improve their application not only in cancer treatment but also in various clinical and industrial settings. This combined approach retains all the advantages of nsPEF-reduced muscle excitation, intracellular modulation, lower energy requirements, and improved field uniformity - while also decreasing the electroporation threshold through the complementary action of MHz bursts and nisin. Together, these effects establish a synergy that could facilitate the translation of nsPEF-assisted ECT into clinical practice by enabling simpler, safer, and more cost-effective pulse generators.

## 2 Materials and methods

### 2.1 Cell culture

The murine mammary carcinoma cell line 4T1 (ATCC^®^ CRL-2539) was cultured in RPMI 1640 medium supplemented with 10% fetal bovine serum (FBS), L-glutamine, 25 mM HEPES, 100 U/mL penicillin, and 100 µg/mL streptomycin (all from Gibco, Thermo Fisher Scientific, New York, NY, USA). Cells were maintained as monolayers at 37°C in a humidified atmosphere containing 5% CO_2_ and used for experiments upon reaching 80% confluency. All experiments were performed using mycoplasma-free cells. The 4T1 cell line was routinely tested for mycoplasma contamination using the MycoBlue^®^ Mycoplasma Detection Kit (Vazyme, Nanjing, China). Unless otherwise specified on the day of the experiment, cells were enzymatically detached using trypsin-EDTA (Thermo Fisher Scientific, Grand Island, NY, USA), collected by centrifugation, and resuspended in electroporation (EP) buffer (242 mM sucrose, 5.5 mM Na_2_HPO_4_, 3 mM NaH_2_PO_4_, 1.7 mM MgCl_2_; pH 7.1) with or without the addition of nisin.

### 2.2 Nisin incubation

**Figure 2:**
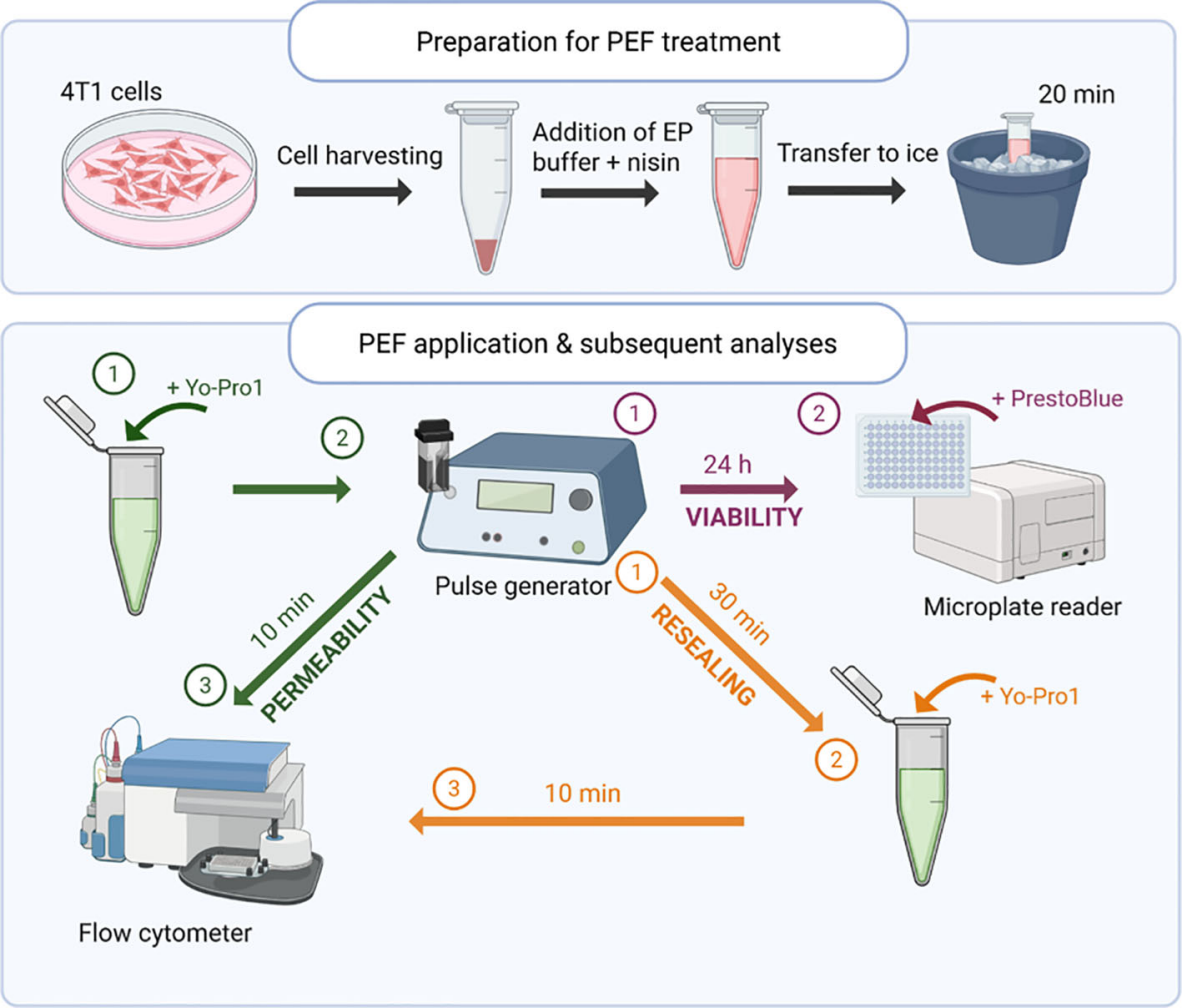
Schematic representation of the experimental workflow. 4T1 cells were incubated with or without nisin Z, subjected to pulsed electric field (PEF) treatment, and subsequently analyzed for membrane permeability, resealing, and viability. Flow cytometry and metabolic assays were used to assess treatment effects at defined time points. Created in https://BioRender.com.

A stock solution of nisin Z (Cas No. 137061-46-2, Cat. no. T76264, TargetMol, Linz, Austria) was prepared at a concentration of 0.5 mg/mL in sterile distilled water. Prior to electroporation, cells were incubated with 50 µg/mL nisin in EP buffer for 20 minutes on ice. The control cells were treated identically, except distilled water without nisin was added to cell suspension to achieve the same dilution. Following incubation, the cell suspension was transferred into electroporation cuvettes for pulse application. The experimental protocol is presented in **Figure 2**.

### 2.3 Electroporation setup and parameters

Pulsed electric field was delivered to cells with a custom-made square-wave high-voltage pulse generator (VilniusTECH, Vilnius, Lithuania) (51). Unless otherwise specified, pulses were applied to the cells in commercially available electroporation cuvettes with a 1 mm electrode gap (Biorad, Hercules, CA, USA), using a sample volume of 50 µL per cuvette. Voltages ranging from 0.06 to 1.2 kV were applied to the cuvette, generating electric fields between 0.6 and 12 kV/cm, respectively. For microsecond pulsed electric fields (µsPEF), the protocols used were: 0.6–1.5 kV/cm, 100 µs pulse duration, 8 pulses, delivered at 1 Hz. Nanosecond pulsed electric fields (nsPEF) consisted of 100 pulses (2–12 kV/cm, 300 ns pulse duration), applied at frequencies of either 10 kHz or 1 MHz. 10 kHz (100 µs interpulse - full membrane relaxation) and 1 MHz (1 µs interpulse - near the PM charging time) were chosen to verify if the partial charge accumulation in high pulse repetition frequency bursts impacts the permeabilization threshold in the nisin’s presence.

### 2.4 Viability assay

Cell viability was assessed 24 hours post-treatment using the PrestoBlue^®^ Cell Viability Reagent (Thermo Fisher Scientific, Grand Island, NY, USA). Cells at a concentration of 2 × 10^6^ cells/mL were exposed to PEFs and transferred into a 96-well flat-bottom plate (TPP, Trasadingen, Switzerland). After a 10-minute incubation at room temperature, growth medium was added to each well to a final volume of 200 µL, and the plate was incubated for 24 hours at 37°C in a 5% CO_2_ atmosphere. The next day, wells were gently rinsed twice with 150 µL of phosphate-buffered saline (PBS) (Gibco, Thermo Fisher Scientific, Waltham, MA, USA). Each well was then filled with 150 µL of PBS and 5 µL of PrestoBlue^®^ reagent. After a 2-hour incubation, metabolic activity was measured using a Synergy 2 microplate reader equipped with Gen5 software (PN 5321002, BioTek, Shoreline, WA, USA), with excitation at 540/20 nm and emission at 620/40 nm.

### 2.5 Cell permeabilization

**Figure 3:**
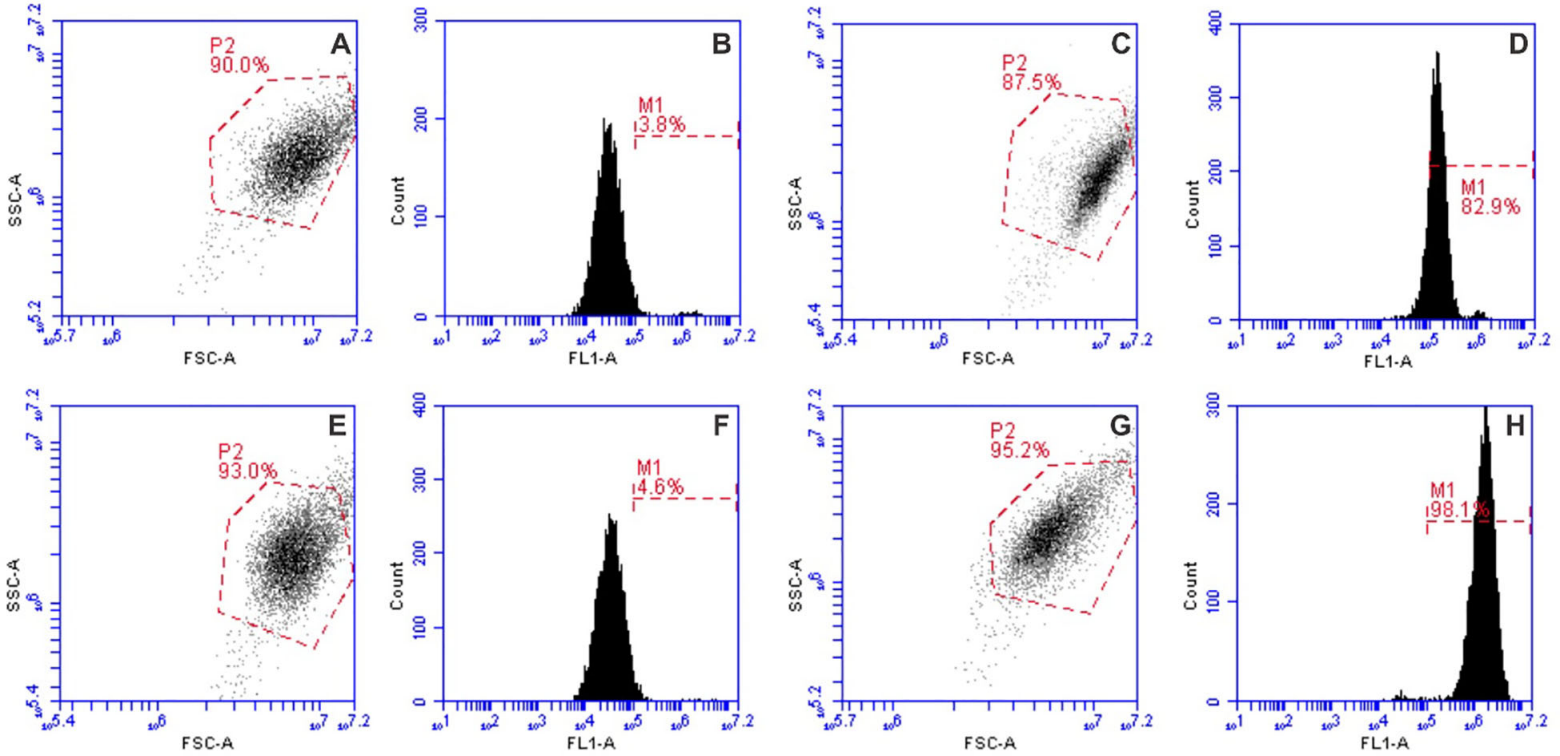
Flow cytometry gating strategy of YP dye, where **(A, B)** and **(C, D)** graph pairs are the untreated control and 1.2 kV/cm × 100 µs × 8 electroporation protocol, respectively; **(E, F)** and **(G, H)** graph pairs are the nisin-treated and 1.2 kV/cm × 100 µs × 8 electroporation protocol with nisin, respectively.

Cell permeabilization induced by electroporation in 4T1 cells (2 × 10^6^ cells/mL) was assessed using the green fluorescent dye YO-PRO-1 (YP, Sigma–Aldrich, St. Louis, MO, USA). Following incubation with nisin, cells were mixed with YP dye to achieve a final concentration of 1 µM and transferred into EP cuvettes. After PEF treatment, the cell suspensions were transferred to FACS FlowTubes (Corning, New York, USA) or 3.5 mL SampleTubes (Sysmex Partec GmbH, Goerlitz, Germany) combined with the Small Volume Sample Tubes (Sysmex). Following a 10-minute incubation at room temperature, 150 µL of a 0.9% NaCl solution (Chempur, Piekary Śląskie, Poland) was added to each tube. Untreated control samples were used to establish gating parameters. Samples were analyzed using a BD Accuri C6 flow cytometer (BD Biosciences, San Jose, CA, USA) or CyFlow^®^ Cube 6 (Sysmex). YP fluorescence was detected in Channel FL1, with excitation at 491 nm and emission collected at 533/30 nm bandpass filter (BPF). Flow cytometry gating for YO-PRO-1 fluorescence was performed as shown in **Figure 3**, allowing discrimination between permeabilized and non-permeabilized cells across control, nisin-treated, and electroporated samples.

### 2.6 Resealing assay

Similar to the permeability assay, cell membrane resealing was evaluated using the green fluorescent dye YO-PRO-1. Following incubation with nisin Z, cells were electroporated and immediately transferred to FACS FlowTubes, where they were incubated at room temperature for 30 minutes to allow membrane resealing. Subsequently, YO-PRO-1 was added to a final concentration of 1 µM. After a 10-minute incubation at room temperature, 200 µL of 0.9% NaCl solution was added to each tube. YP fluorescence was measured using the same flow cytometry protocol as described for the permeability assay.

### 2.7 Morphological assessment

For the assessment of cell morphology, instead of 1 mm cuvettes, a custom-made tweezer electrode with 1 mm gap was used to apply µsPEF and nsPEF. For the experiments, cells were harvested from culture flasks and seeded on ibidiTreat µ-dishes (Ibidi, Gräfelfing, Germany): 2.4 × 10^4^ cells were seeded in each dish 48 hours prior to experiment. On the day of the experiment, cells were incubated with an EP buffer with or without nisin for 20 minutes at 4°C and then subjected to PEF. Next, the EP buffer was replaced with a fresh RPMI medium and the holotomographic imaging was performed 10 minutes after PEF application using a 3D Cell Explorer (Nanolive, Tolochenaz, Switzerland). The data processing and 3D refractive index distribution were determined using a commercially available STEVE software (Nanolive).

### 2.8 Statistical analysis

Statistical analysis was performed using one-way analysis of variance (ANOVA), with a significance threshold set at p < 0.05. When ANOVA indicated significant differences among groups, *post hoc* comparisons were conducted using the Tukey HSD multiple comparison test (p < 0.05 considered statistically significant). All data presented in graphs were analyzed and presented using OriginPro software (version 18.0, OriginLab, Northampton, MA, USA). To enhance clarity, graph colors were standardized throughout the paper: nisin-alone treatments are represented in blue, PEF treatments without nisin in green, and combined PEF and nisin treatments in red. Each data point represents the mean of at least three independent experiments and is expressed as mean ± standard deviation. The EF_50_ values were calculated based on a nonlinear regression dose-response curve, using a GraphPad Prism software (Boston, Massachusetts USA), version 10.5.0 for Windows.

## 3 Results

### 3.1 Determination of non-toxic nisin concentration for combined electroporation treatment

**Figure 4:**
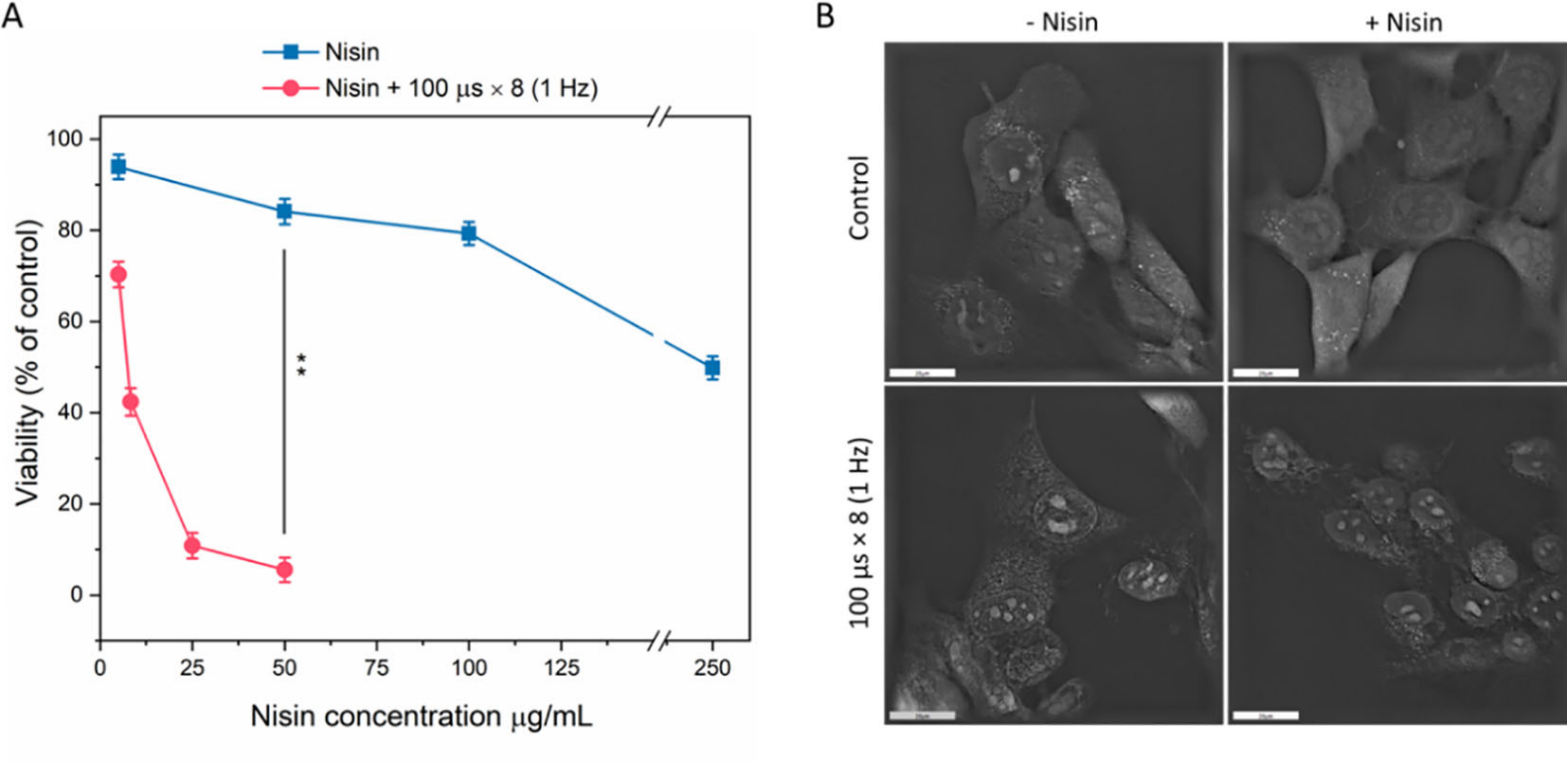
Cell viability **(A)** and morphology **(B)** after treatment with nisin Z alone or combined with pulsed electric field (PEF: 1.2 kV/cm, 8 pulses, 100 µs, 1 Hz). Viability (% of control) was assessed following exposure to increasing nisin Z concentrations (0–250 µg/mL), with (red circles) or without (blue squares) PEF. Data shown as mean ± SD (**p < 0.01). Cell morphology was assessed with holotomographic microscopy on adherent cells exposed to 50 µg/mL nisin and PEF. Scale bar: 20 µm.

The first step of the study was to determine a non-toxic concentration of nisin that would remain biologically active when combined with electroporation, while at the same time remaining predominantly non-toxic for the cells when EP is not involved. The results are summarized in **Figure 4**.

Based on cell viability assays, a concentration of 50 µg/mL (14.9 µM) was selected, as it did not cause a considerable decline in viability in regard to non-treated control (**Figure 4A**). However, when combined with µsPEF (according to the standard ESOPE protocol), at 1.2 kV/cm cell viability decreased from ~84% to ~5.6% (p < 0.001, n = 3), indicating a strong synergistic cytotoxic effect. When the same PEFs were applied to adherent cells, pronounced membrane irregularities were observed even in the absence of nisin (**Figure 4B**). However, when nisin was added to the EP buffer, cells showed morphological alterations, including loss of membrane integrity. Some cells appeared swollen with irregular contours and disrupted internal structure, consistent with membrane destabilization and loss of cellular homeostasis.

### 3.2 The impact of nisin on permeabilization and resealing following the ESOPE protocol

**Figure 5:**
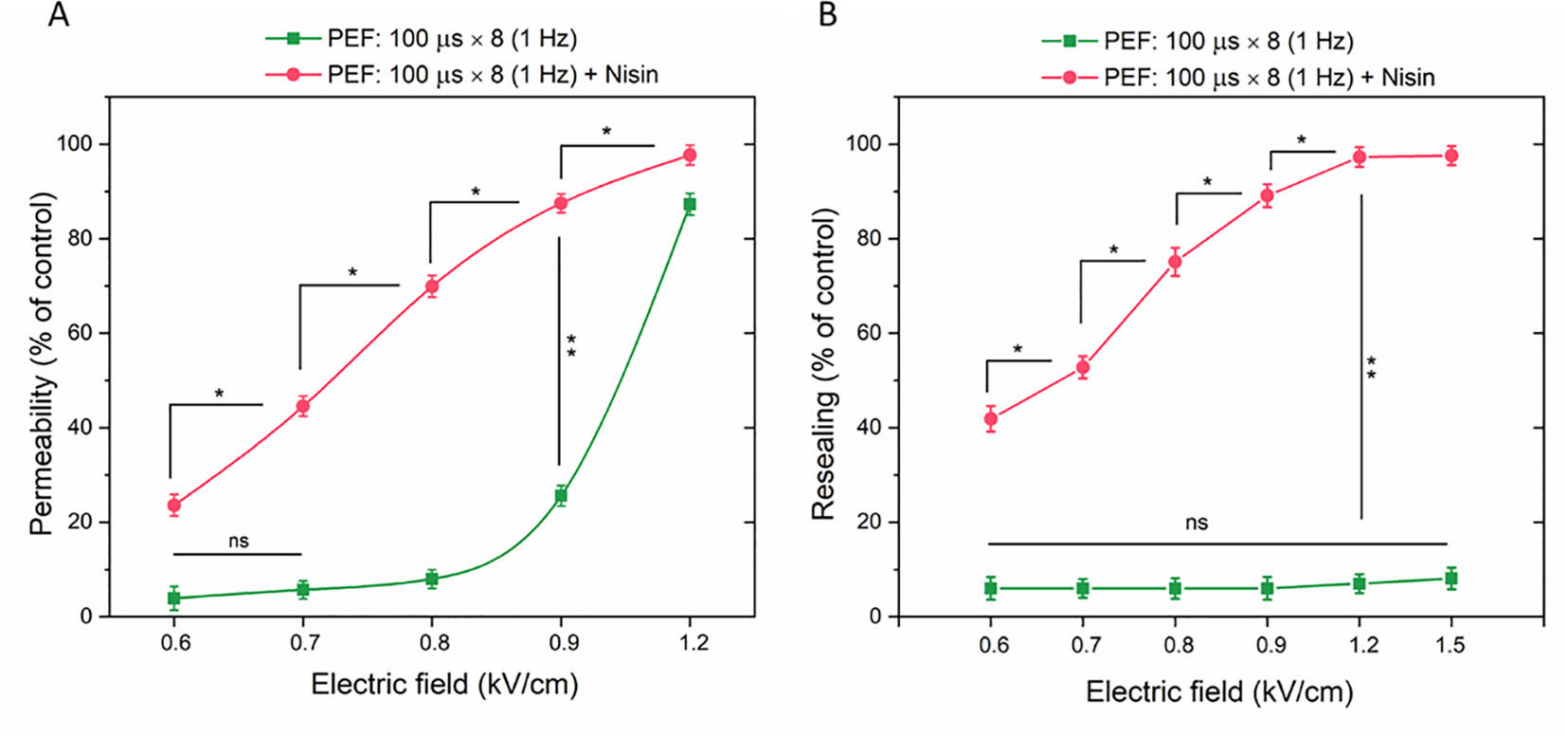
The permeabilization **(A)** and resealing **(B)** of 4T1 cells as a function of YP fluorescence in cells subjected to microsecond pulses (ESOPE protocol) with or without nisin. Data shown as mean ± SD (*p < 0.05, **p < 0.01, ns, non-significant). For better clarity, only selected statistically significant differences are highlighted on the graph.

The selected subtoxic nisin concentration of 50 µg/ml was used to sensitize 4T1 cells to PEF. Cytometric measurements of YO-PRO-1 dye fluorescence demonstrated that incorporating nisin into the ESOPE protocol significantly enhanced the permeabilization efficacy of µsPEF, allowing more dye to enter through electroporated membrane (**Figure 5A**). The most pronounced differences between groups with and without nisin were detected for the electric field strength of 0.8 kV/cm (70% vs 8%, respectively) and for 0.9 kV/cm (~88% vs 26%, respectively).

Similar measurements were conducted in cells subjected to µsPEF with or without nisin, with the YP dye added 30 minutes after the electroporation protocol. This approach allowed the fluorescent signal to be detected only in cells that failed to reseal after electroporation. Within the tested range (0.6 – 1.5 kV/cm), none of the electric field strengths significantly affected the cell resealing process (**Figure 5B**). When nisin was included in the protocol, 42% of cell population was not resealed even with the lowest tested electric field strength. At 1.2 kV/cm, almost entire cell population was permeable to YP 30 minutes after PEF application. Notably, for lower tested electric field parameters (0.6 and 0.7 kV/cm) the percentage of YO-PRO-1-positive cells was higher in Resealing population vs Permeability population, demonstrating that cell permeability was increased even 40 minutes after the combined PEF + Nisin treatment.

### 3.3 The impact of nisin on permeabilization and resealing following nsPEF at various pulse frequencies

**Figure 6:**
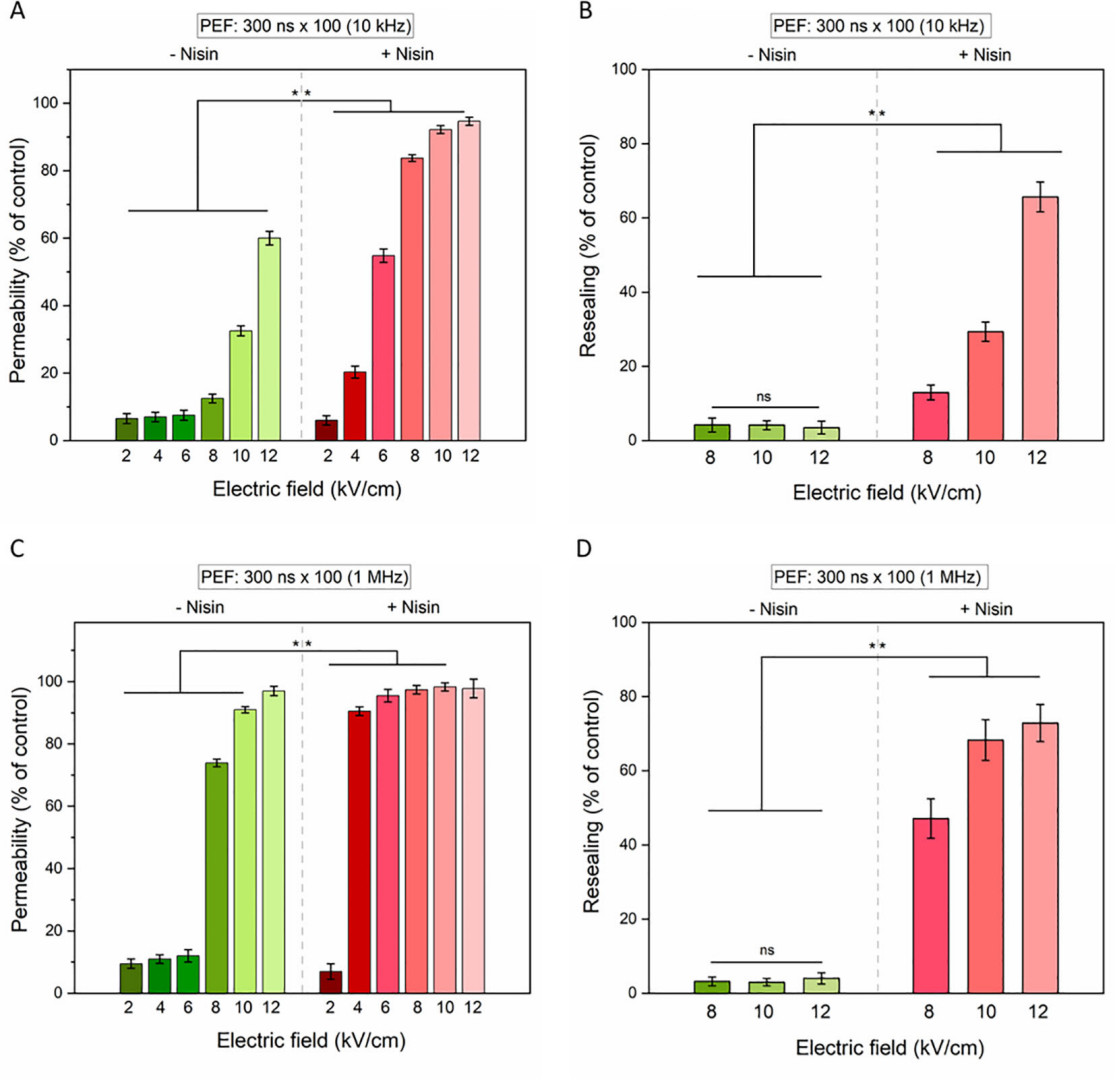
The permeabilization (left column) and resealing (right column) of 4T1 cells as measured by YO-PRO-1 (YP) fluorescence in cells subjected to 100 × 300-nanosecond pulses with electric field intensities ranging from 2 to 12 kV/cm and pulse frequencies of 10 Hz **(A, B)** or 1 MHz **(C, D)**. Data shown as mean ± SD (**p < 0.01, ns, non-significant).

Similar to the ESOPE protocol, nisin markedly augmented the bioeffects of PEFs in 4T1 cells exposed to nsPEFs for both tested pulse frequencies 10 kHz (**Figures 6A, B**) and 1 MHz (**Figures 6C, D**).

At both frequencies, membrane permeability and resealing impairment increased with rising electric field intensities. For the 10 kHz condition (**Figure 6A**), a synergistic effect between PEF and nisin was particularly evident at electric fields ≥8 kV/cm, with a significant enhancement in membrane permeability, as indicated by increased YO-PRO-1 uptake (**Figure 6B**). In contrast, under the 1 MHz condition (**Figure 6C**), statistically significant differences between treatments with and without nisin were observed primarily at lower electric field strengths (≥8 kV/cm). At higher field strengths, nearly the entire cell population was permeabilized even in the absence of nisin, minimizing the observed differential effect. Notably, cells exposed to high electric fields without nisin demonstrated an ability to reseal their membranes over time, as reflected by a reduced proportion of cells stained with YO-PRO-1–30 minutes after PEF protocol. Conversely to this, the addition of nisin resulted in sustained membrane permeabilization and impaired resealing capacity (**Figure 6D**), underscoring the potentiating effect of nisin on PEF-induced cytotoxicity.

### 3.4 The impact of nisin on viability and morphology following various PEFs protocols

**Figure 7:**
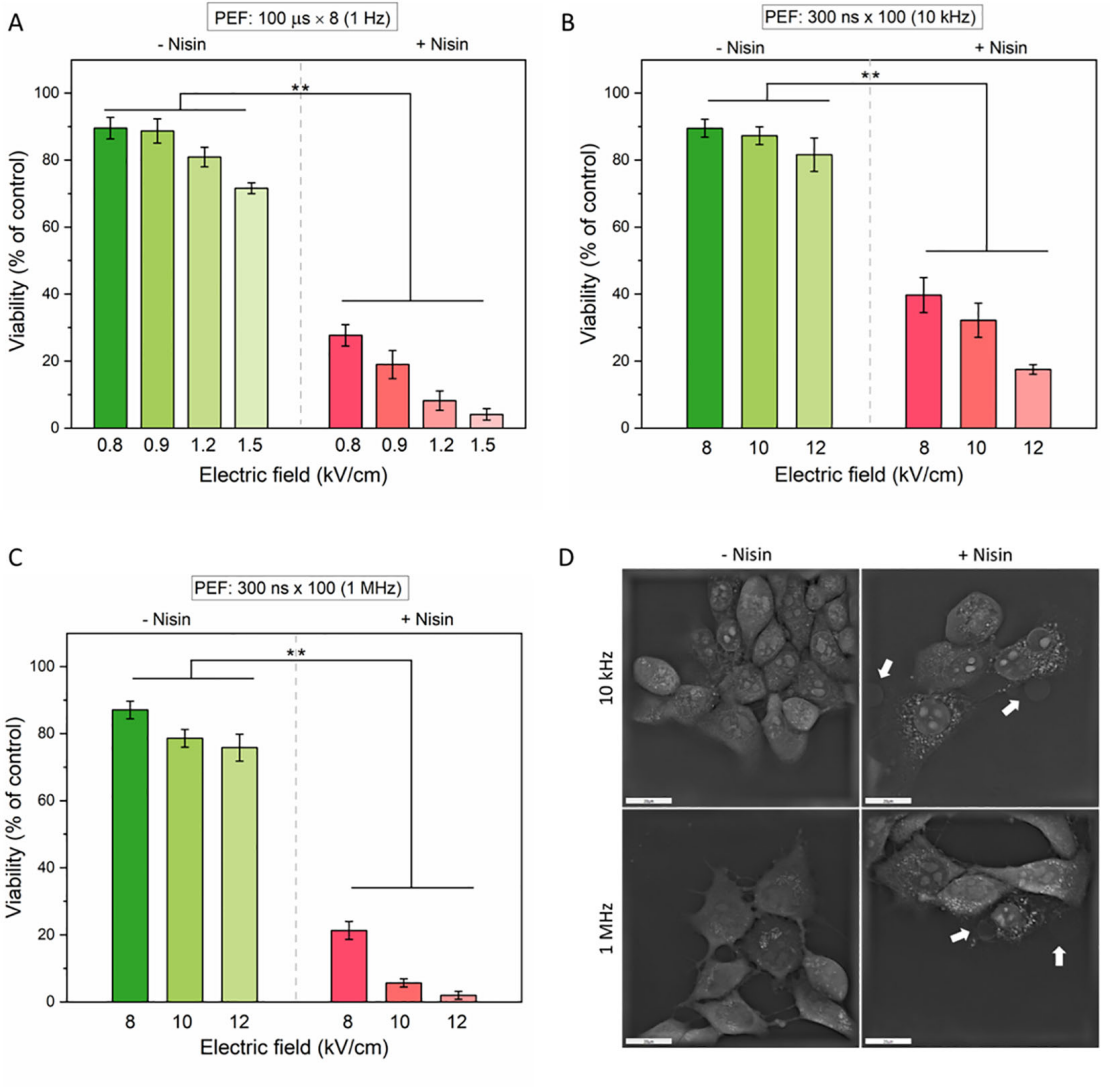
The effect of pulsed electric fields (PEFs) with or without nisin on cell viability **(A–C)** and morphology **(D)**. Cells were exposed to increasing electric field strengths under three different pulse conditions: **(A)** Microsecond pulses: 100 μs × 8 at 1 Hz; **(B)** Nanosecond pulses (nsPEF): 300 ns × 100 at 10 kHz; **(C)** Nanosecond pulses: 300 ns × 100 at 1 MHz. Data shown as mean ± SD (**p< 0.01). Cell morphology was assessed with holotomographic microscopy on adherent cells exposed to 50 µg/mL nisin and nsPEF (8 kV/cm, 300 ns × 100 at 10 kHz or 1 MHz). Cell blebbing was marked with white arrows. Scale bar: 20 µm.

Previous experiments on 4T1 cell membrane resealing demonstrated that the addition of nisin significantly impaired the resealing capacity following PEF treatment. However, these experiments did not clarify whether nisin prolongs the existence of membrane pores or if the increased permeability leads to irreversible damage and cell death. To address this, the viability of 4T1 cells was assessed 24 hours after exposure to various PEF protocols, both in the presence and absence of nisin (**Figures 7A–C**). Additionally, cell morphology was examined in adherent cells right after pulse delivery (**Figure 7D**).

In all tested PEF modalities, cell viability decreased with increasing electric field strength, and the addition of nisin significantly enhanced cytotoxicity. For µsPEF, a moderate reduction in cell viability was noted (from ~90% to ~75%) in the electric field range of 0.8 – 1.5 kV/cm (**Figure 7A**). However, with nisin, viability dropped sharply at even the lowest field, reaching below 5% at 1.5 kV/cm. A similar pattern was evident for nsPEF protocols. At 10 kHz frequency (**Figure 7B**), cell viability dropped below 20% in the presence of nisin. In contrast, 1 MHz stimulation (**Figure 7C**) produced an even more pronounced cytotoxic effect, reducing viability to below 5% at 12 kV/cm. These findings underscore the potentiating effect of nisin on PEF-induced cell death, particularly at higher field strengths and pulse frequencies. To confirm that the observed nisin-nsPEF synergy occurs not only in cells in suspension, we performed a visualization of adherent cells 10 minutes after PEF application (**Figure 7D**). In both tested nsPEF modalities, we observed a loss of the membrane integrity and subsequent formation of bubbles on the cell surface, consistent with cytoplasmic leakage or cell blebbing. Notably, the morphology of cells subjected to nsPEF differed from the morphology of cells treated with ESOPE protocol (see **Figure 4B** for comparison). Cell membranes in samples exposed to µsPEF alone (without nisin) appeared to be more visibly disrupted than those treated with nsPEF. When nisin was present in the electroporation buffer, all PEF-treated cells showed membrane defects; however, the formation of bubbles was observed exclusively in cells treated with pulses of nanosecond range.

### 3.5 The impact of nisin on the threshold electric field required to evoke a biological effect in 50% of the cell population (EF_50_).

To summarize various biological effects of nisin addition and to facilitate the comparison between tested PEF modalities, the electric field intensities allowing to affect 50% of the population (EF_50_) were calculated for each experiment separately: permeabilization, resealing and viability (**Table 1**).

**Table 1:** EF50 values (electric field strength required to affect 50% of cells) for membrane permeabilization, resealing, and viability under different pulsed electric field (PEF) modalities, with and without the presence of nisin.

PEF modality	Nisin presence (without nisin: -, with nisin: +)	EF_50_ permeabilization (kV/cm)	EF_50_ resealing (kV/cm)	EF_50_ viability (kV/cm)
µsPEF, 100 μs × 8 at 1 Hz	–	1.00	>> 1.50	> 1.50
	+	0.71	0.66	0.61
nsPEF, 300 ns × 100 at 10 kHz	–	11.27	>>12.000	>> 12.00
	+	5.60	11.07	6.93
nsPEF, 300 ns × 100 at 1 MHz	–	7.25	>>12.00	>> 12.00
	+	2.18	8.15	6.57

In some experiments, the calculated EF50 values significantly exceeded the experimental range; these values were marked as ‘>>’, indicating that they lie much higher than the highest field strength tested.

Nisin consistently reduced the EF_50_ for permeabilization, resealing and viability across all tested protocols. The most pronounced effects on membrane permeability after nisin addition were detected for nsPEFs of 1 MHz, with 3.33-fold reduction of the EF_50_ value with regard to the nisin-free samples. As without nisin, almost entire cell population was able to reseal, even with the highest tested PEF parameters, it was not possible to determine exact reduction in EF_50_ values between nisin-treated and nisin-free samples. However, a significant reduction in the electric field affecting the resealing ability of 50% of population was noted for all the tested PEF modalities. The further viability studies indicate that the differences in cell resealing stem from the irreversible cell damage rather than delayed cell resealing. In terms of viability, all tested PEF modalities were enhanced by nisin addition, reaching the 4.22, 3.87 and 3.4-fold reduction in EF_50_ Viability in samples treated with 10 Hz nsPEFs, µsPEFs and 1 MHz nsPEFs, respectively. These findings support the hypothesis that nisin is a promising agent that can be used in ECT treatment and at the same time can be part of the strategy to lower the PEF amplitude requirements during nsPEF application, which is not the case for conventional ECT agents.

## 4 Discussion

This study demonstrates that the antimicrobial peptide nisin significantly enhances the bioeffects of pulsed electric fields (PEFs), both microsecond (µsPEF) and nanosecond (nsPEF), in murine 4T1 cancer cells. The concentration of 50 µg/mL nisin, identified as non-toxic when used alone, was shown to produce a strong synergistic effect when combined with µsPEF, decreasing cell viability from ~84% to ~5.6%. The combined treatment produced a significantly greater reduction in cell viability than either modality alone, indicating that nisin enhances the cytotoxic impact of PEF beyond additive effects. This finding supports the concept that membrane permeabilization facilitates nisin’s intracellular action and strengthens the rationale for its use as an adjuvant or potential standalone drug in electrochemotherapy. The observed synergy at various sub-toxic nisin concentrations also suggests that therapeutic efficacy might be achieved with lower drug doses, potentially minimizing systemic side effects.

Given that nisin is an FDA-approved food preservative, there has been natural interest in combining it with electroporation for enhanced microbial control. As early as in 1999 Calderón-Miranda et al. studied the sensitization of *Listeria innocua* to nisin liquid whole egg and skimmed milk following PEF (52, 53). While additive effects were observed at lower intensities, a synergistic effect emerged with higher electric field strength, pulse numbers, and nisin concentrations (53). Later, the favorable effect of PEF and nisin (alone or encapsulated in nanoparticles) was demonstrated also against *Staphylococcus aureus* (54), *Salmonella typhimurium* (55), *Bacillus cereus* (56), and *Escherichia coli* (57). Numerous studies demonstrated that Lipid II acts as a key docking molecule, significantly enhancing nisin’s pore stability (15, 20, 58, 59). However, studies on liposomes indicate that the presence of Lipid II is not essential for inducing nisin-related membrane permeabilization (19, 60). The idea to combine permeabilizing properties of nisin with the electroporation technique has been explored before in bacterial and artificial membranes. For example, the study by Yi et al. (2013) demonstrates that incorporating nisin into liposomal membranes significantly enhances pore formation and stabilization during EP, facilitating efficient release of encapsulated cargo (5–6 carboxyfluorescein dye) (61). To our knowledge, up to now, there has been no study demonstrating the sensitization of cancer cells to electroporation following nisin exposure. Permeabilization experiments using YO-PRO-1 highlighted nisin’s potentiating effect. Cells treated with nisin + PEF showed markedly increased membrane permeability compared to PEF alone, particularly at intermediate electric field strengths (e.g., 0.8–0.9 kV/cm). This suggests that nisin may be involved in the formation or stabilization of electropores. However, the increase in the YO-PRO-1 uptake in cells even 40 minutes after pulse delivery supports the notion that PEF and nisin synergy translates into irreversible damage and cell death, rather than reversible permeability changes alone.

Although synergistic effects of nisin and PEF were apparent for both - µsPEF and nsPEF, some differences were spotted in the mode of action when pulses were delivered to the adherent cells. With nsPEFs a formation of bubbles on the cell surface was observed in the nisin’s presence. The membrane protrusions seen after nsPEF with nisin likely result from the combined effects of ultrashort pulses and peptide-induced membrane destabilization. Although they resemble blebs, they more likely represent localized swelling or cytoplasmic leakage caused by Ca^2+^-mediated stress often accompanying nsPEF application (62) or are attributed to nisin interaction with the bilayer lipids. Thus, these bubbles are best interpreted as evidence of membrane destabilization, rather than classical apoptotic blebbing. Notably, these conclusions are based solely on qualitative observations; quantitative image analysis of adherent cells exposed to µsPEF and nsPEF across a range of field amplitudes is still required to delineate nisin’s effects in this experimental setup.

Strikingly, the degree of cytotoxicity enhancement achieved with nisin mirrors or even exceeds the clinical benchmarks from ECT. The level of nisin cytotoxicity enhancement is comparable to that achieved with clinically approved ECT’s cytostatic cisplatin. For instance, the IC_50_ of cisplatin was decreased by 3− to 13−fold when combined with electroporation, compared to cisplatin treatment alone (63). In our study, nisin IC_50_ towards 4T1 cells was decreased ~20-fold. This parallel highlights nisin’s potential to be incorporated into ECT protocols, especially in scenarios where alternative or lower-toxicity agents are desired. Importantly, the observed interaction between nisin and pulsed electric fields was bidirectional. Not only did nisin exhibit enhanced cytotoxicity when combined with PEFs, but its presence also lowered the electric field intensity required to achieve effective electroporation outcomes. Although exact EF_50_ values for resealing could not be determined in some conditions because most untreated cells were able to recover even after high-voltage exposure, a clear downward shift in the electric field affecting 50% of the population was observed for all tested protocols. This reduction indicates that nisin sensitizes cells to electric-field exposure, minimizing energy input.

Importantly, the frequency-dependent effects observed under nsPEF conditions suggest that higher-frequency fields (1 MHz) produce more widespread electroporation, leaving less room for nisin to exert an additive effect at higher field strengths. However, at moderate intensities, the presence of nisin significantly lowered the threshold required to achieve permeabilization or cytotoxicity (reflected in EF_50_ values), which could be leveraged therapeutically to minimize the need for high-voltage application and reduce off-target effects.

The main limitations to the practical application of nisin are its variable activity under different conditions and its instability in biological environments. The activity of nisin has been shown to be significantly influenced by several factors, including the phospholipid composition of the target membrane (64), the ionization state of its histidine residues (19), and the peptide concentration (17). Variations in membrane lipid composition can modulate nisin’s ability to insert and form pores, while the protonation state of histidine residues - dependent on pH - affects its structural conformation and membrane interactions. At low pH (~2.0), nisin can withstand autoclaving without losing its activity (65). Furthermore, nisin exhibits concentration-dependent behavior, where pore formation and antimicrobial activity increase with higher peptide levels, often showing threshold effects characteristic of cooperative binding and membrane disruption. Therefore, careful evaluation using various cell types is needed to fully reveal the therapeutic potential of nisin in conjunction with EP and ECT. Importantly, the study did not determine, if the increased permeability is attributed to the increased number of pores or the delayed cell resealing. Except for the pore formation and their lifespan, nisin may also influence membrane repair kinetics, lipid organization, or other processes affecting dye uptake and viability. Therefore, further studies using spatially resolved approaches are needed to establish whether the effect reflects a lowered electroporation threshold or altered resealing dynamics.

## 5 Conclusion

We demonstrated that nisin markedly enhances the cytotoxic effects of pulsed electric fields across a range of electric field intensities, pulse durations, and frequencies. Collectively, the results suggest that nisin can act as a potent sensitizer in electroporation-based therapies by lowering the effective thresholds for membrane disruption. This could improve the applicability and controllability of PEFs, particularly when using short pulses that typically require high voltages. Furthermore, nisin shows potential as an electrochemotherapy agent in its own right. Recognized as safe by the WHO, ESFA and FDA, nisin emerges as a novel, natural ECT candidate. Future studies should explore the molecular mechanisms underlying the increased permeability and decreased viability, possibly involving nisin’s interactions with membrane lipids or cytoskeletal elements, as well as a more in-depth investigation of the type of cell death. It will be necessary to confirm nisin’s efficacy against different types of cancer cells and, in the context of ECT, to verify its activity in the presence of commonly used drugs. Additionally, *in vivo* validation and investigation of tissue-specific effects will be essential for translating these findings into clinical applications.

## Data Availability

The data is publicly available on the Figshare platform. ID: 10.6084/m9.figshare.30610217.
